# Deviation from normative brain development is associated with symptom severity in autism spectrum disorder

**DOI:** 10.1186/s13229-019-0301-5

**Published:** 2019-12-11

**Authors:** Birkan Tunç, Lisa D. Yankowitz, Drew Parker, Jacob A. Alappatt, Juhi Pandey, Robert T. Schultz, Ragini Verma

**Affiliations:** 1 Center for Autism Research, The Children’s Hospital of Philadelphia, Philadelphia, PA 19104 USA; 2 Department of Biomedical and Health Informatics, The Children’s Hospital of Philadelphia, Philadelphia, PA 19104 USA; 3 Department of Psychiatry, University of Pennsylvania, Philadelphia, PA 19104 USA; 4 Center for Biomedical Image Computing and Analytics, University of Pennsylvania, Philadelphia, PA 19104 USA; 5 Department of Psychology, University of Pennsylvania, Philadelphia, PA 19104 USA; 6 DiCIPHR (Diffusion and Connectomics in Precision Healthcare Research) Lab, Department of Radiology, University of Pennsylvania, Philadelphia, PA 19104 USA; 7 Department of Pediatrics, University of Pennsylvania, Philadelphia, PA 19104 USA

**Keywords:** Autism, Brain development, Heterogeneity, Symptom severity, Machine learning, Normative modeling

## Abstract

**Background:**

Autism spectrum disorder (ASD) is a heterogeneous neurodevelopmental condition. The degree to which the brain development in ASD deviates from typical brain development, and how this deviation relates to observed behavioral outcomes at the individual level are not well-studied. We hypothesize that the degree of deviation from typical brain development of an individual with ASD would relate to observed symptom severity.

**Methods:**

The developmental changes in anatomical (cortical thickness, surface area, and volume) and diffusion metrics (fractional anisotropy and apparent diffusion coefficient) were compared between a sample of ASD (*n* = 247) and typically developing children (TDC) (*n* = 220) aged 6–25. Machine learning was used to predict age (brain age) from these metrics in the TDC sample, to define a normative model of brain development. This model was then used to compute brain age in the ASD sample. The difference between chronological age and brain age was considered a developmental deviation index (DDI), which was then correlated with ASD symptom severity.

**Results:**

Machine learning model trained on all five metrics accurately predicted age in the TDC (*r* = 0.88) and the ASD (*r* = 0.85) samples, with dominant contributions to the model from the diffusion metrics. Within the ASD group, the DDI derived from fractional anisotropy was correlated with ASD symptom severity (*r* = − 0.2), such that individuals with the most advanced brain age showing the lowest severity, and individuals with the most delayed brain age showing the highest severity.

**Limitations:**

This work investigated only linear relationships between five specific brain metrics and only one measure of ASD symptom severity in a limited age range. Reported effect sizes are moderate. Further work is needed to investigate developmental differences in other age ranges, other aspects of behavior, other neurobiological measures, and in an independent sample before results can be clinically applicable.

**Conclusions:**

Findings demonstrate that the degree of deviation from typical brain development relates to ASD symptom severity, partially accounting for the observed heterogeneity in ASD. Our approach enables characterization of each individual with reference to normative brain development and identification of distinct developmental subtypes, facilitating a better understanding of developmental heterogeneity in ASD.

## Background

Autism spectrum disorder (ASD) is a heterogeneous neurodevelopmental condition associated with atypical trajectories of brain anatomy, function, and connectivity, distinguishing it from typical development [1–8]. Although the exact details of developmental changes in ASD vary across individual studies, one fact is well established in the literature: ASD has a complex and dynamic neurobiological mechanism(s), with the disorder-related changes in the brain showing variations across ages [9, 10]. For example, head circumference and brain imaging data suggest that individuals later diagnosed with ASD have typical brain volume from birth through 6 months of age [11, 12]. This is followed by a period of accelerated growth, resulting in larger brains by age two [13, 14]. Evidence regarding brain volume differences in older children and adolescents with ASD is mixed, although differences in brain volume may be smaller than in toddlerhood [15–17]. Thus, consideration of the development is crucial to understanding the neurobiology of ASD.

The differential development of ASD pathology suggests that the *expected* neuroanatomical features of individuals with ASD should be adjusted based on their ages; that is, the exact nature of atypicality in ASD and the direction of deviation from typicality (e.g., bigger brains or smaller brains) may change with age. Furthermore, individuals with ASD also show variations within their age groups and follow different developmental trajectories [18, 19], and even make shifts between various possible trajectories at different ages [20]. Therefore, a comprehensive understating of heterogeneous behavioral impairments in ASD requires studies that can elucidate developmental blueprints of the brain and identify distinct developmental subtypes of ASD associated with divergent behavioral profiles.

The ASD-related developmental changes on the anatomical features of the brain such as volume, cortical surface area, and cortical thickness have been extensively studied [10, 13, 21–23]. For example, the presence of atypical brain enlargement in infancy and early childhood is well documented [14, 23–25]. One aspect of brain development that needs to be further investigated is the interaction between brain anatomy and other neurobiological features related to brain connectivity (e.g., white matter tissue characteristics). In the last decade, brain connectivity alterations (e.g., disruption in diffusion metrics) have emerged as candidate biomarkers in [26–34], owing to recent advances in diffusion MRI (dMRI) [35, 36]. While there exist many neurobiological findings on diffusion-related alterations in ASD, their developmental characteristics remain unclear. Even less is known about how ASD-related developmental differences, both anatomical and diffusion-related, are linked to heterogeneous behavioral profiles.

A comprehensive understanding of developmental heterogeneity in ASD necessitates insights into typical brain development and investigations on individual-level deviations from typical development. Recent normative modeling techniques [37] can facilitate such investigations by modeling brain maturation using a healthy sample, and then quantifying individual differences from this normative model. Within this framework, disorders are conceptualized as extreme values of quantitative biological measures (e.g., neuroimaging metrics) or deviations from normative functioning, enabling a dimensional analysis to study heterogeneous presentations [37, 38].

Normative modeling techniques use prediction algorithms (e.g., machine learning) to model relationship between biological measures and other clinically relevant variables. Once the prediction algorithm is trained using a healthy sample to define a normative model, it is then applied to a patient sample to quantify deviations of individuals from this normative model. These techniques usually use two alternative approaches regarding the directionality of the relationship between biological measures and other clinically relevant variables. One approach is to predict a given single biological measure (e.g., cortical thickness of a specific brain region) from clinical or demographic variables (e.g., age, sex) [38], whereas in the second approach, the mapping is inverted and a clinical or demographic variable is predicted from multiple biological measures (e.g., cortical thickness across all brain regions) [39]. Both approaches can be utilized in neuroimaging studies and have their own advantages and drawbacks.

The first approach allows identification of regional deviations that may change across individuals [40], providing a clear picture on how a single behavioral outcome can be a result of distinct alteration patterns in the brain [41]. The second approach, mostly known as a *brain age* approach, fuses information from multiple regions to predict the chronological age of individuals, implicitly defining normative maturation patterns. Brain age models, while limiting region-specific interpretations, use multivariate machine learning techniques to better capture complex interactions between brain regions during development [42, 43], as well as the interplay between different modalities such as anatomical and diffusion metrics. This approach yields a summary score of multimodal brain development (brain age) and enables defining an index of deviation for each individual (i.e.*,* difference between brain age and chronological age) [39]. Moreover, one may easily model the relationship between this individual-level index and the behavioral differences related to the disorder.

In this study, within a large dataset of children and young adults (*N* = 467, age = 6–25 years, 247 with ASD), we used brain age approach explained above and developed an individual-level measure of multimodal brain development, called *developmental deviation index* (DDI), to parse behavioral heterogeneity in ASD. In order to define a normative model of brain development, we investigated developmental changes in anatomical (cortical volume, cortical surface area, and cortical thickness) and diffusion metrics (fractional anisotropy [FA] and apparent diffusion coefficient [ADC]) of the brain. The normative model, trained using typically developing children (TDC), was then used to study the relationship between individual-level deviation from the normative model and disorder symptom severity in ASD. We hypothesized that the affected participants with a positive DDI (i.e., brain age > chronological age) would manifest less symptom severity compared with the affected participants with a negative DDI (i.e., brain age < chronological age). Our approach enables characterization of each individual with reference to normative brain development and identification of distinct developmental subtypes, facilitating a better understanding of developmental heterogeneity in ASD. This in turn may lead to improved assessment of developmental or treatment-related change, to targeted and individualized treatment planning, and eventually to a true precision medicine approach in ASD.

## Methods

### Participants

Data collection was performed at the Center for Autism Research (CAR) at Children’s Hospital of Philadelphia (CHOP), from February 2009 to February 2013. Data collection and use was approved by the institutional review board (IRB) at CHOP and the University of Pennsylvania. Individuals with a community diagnosis of an ASD were recruited in part through autismMatch (https://autismmatch.org). Diagnoses were confirmed by the clinical core at CAR, using DSM-IV-TR criteria [44], informed by Autism Diagnostic Observation Schedule (ADOS) [45] and Autism Diagnostic Interview-Revised [46], and expert consensus clinical judgment by two independent psychologists following Collaborative Programs of Excellence in Autism diagnostic guidelines. Children with known genetic conditions associated with ASD were excluded from the study. The symptom severity of participants was assessed using ADOS calibrated severity score (CSS) [47], and the cognitive ability (IQ) was assessed with the Differential Abilities Scale, Second Edition [48]. The details on the dataset (*N* = 467, Age = 6–25 years) are given in Table 1.

**Table 1:** Demographics and clinical profile of participants. With *ADOS*, social affect (SA) and restricted and repetitive behaviors (RRB) scores are listed

Diagnosis	Age Min-Max, Mean (Std)	Sex	IQ Mean	ADOS Mean
TDC (*n* = 220)	6.26–25.63 13.06 (4.12)	Male, 161 Female, 59	Verbal, 114.48 Nonverbal, 108.09 Total, 112.81	SA, 2.05 RRB, 2.50 Total, 1.70
ASD (*n* = 247)	6.36–25.87 12.85 (3.52)	Male, 203 Female, 44	Verbal, 100.63 Nonverbal, 100.24 Total, 100.23	SA, 6.76 RRB, 7.09 Total, 6.85

### Image acquisition and processing

T1-weighted anatomical images were acquired on a Siemens 3 T wide-bore Magnetom Verio Tim scanner with a 12-channel head coil and a Siemens MPRAGE sequence (0.8 × 0.8 × 0.9 mm, TR = 1900, TE = 2.54, flip angle = 9). Images were N3 bias corrected with ANTS [49] and brain extracted with LABEL [50]. Measures of cortical volume, cortical surface area, and cortical thickness were derived using the Freesurfer image analysis suite version 5.3.0 [51], for each region in the Desikan-Killiany cortical atlas [52] (34 cortical regions of interest per hemisphere).

Diffusion characteristics of the brain tissue were assessed by diffusion weighted imaging (DWI). The DWI dataset was acquired in three epochs on the same scanner (Siemens Verio 3 T). In the first epoch, DWI was acquired using a monopolar sequence, with repetition time(TR)/echo time (TE) = 14,000/70 ms. In the second epoch, DTI was acquired at TR/TE = 11,000/75 ms using a monopolar+ sequence. In the third, DTI was acquired at TR/TE = 11,000/76 ms using a monopolar sequence. All data was acquired with an image resolution of 2 × 2 × 2 mm, collecting 30 directions with *b*-value = 1000 s/mm^2^ and one *b* = 0 image. We verified that there were no significant difference between scanning epochs in terms of DTI metrics of fractional anisotropy (FA) and apparent diffusion coefficient (ADC), as well as participant age, IQ, and ADOS severity. The diffusion metrics did not show any significant difference across the acquisition protocols, neither in different tissue types, nor in the whole brain.

In order to quantify the MRI motion, we used the technique proposed in [53]. We used “eddy” from FSL 5.0 to estimate volume-to-volume rotations and translations. These measures were then transformed from radian angles to displacement in millimeters, from which the root mean square displacement was computed.

In the final dataset, 467 participants had at least one imaging modality (anatomical and diffusion) that passed our QA pipelines (see Additional file 1: Section S1 for details). Three hundred forty-nine participants had reliable diffusion metrics, and 415 participants had reliable anatomical metrics. Two hundred sixty-one participants had both diffusion and anatomical metrics acquired during the same session. When training a machine learning model using a single modality, we used all available data for that modality (i.e., 349 for diffusion and 415 for anatomical). For the combined model, we used only 261 participants who had both modalities.

### Multivariate-multimodal analysis using machine learning

We used support vector regression (SVR) [54] for predicting chronological age of participants from diffusion and anatomical metrics. SVR is commonly used in brain imaging studies [39, 55] due to its superiority in interpretation of outcomes, as compared with more complex methods (e.g., deep learning models) that obscure neurobiological interpretations on inner workings of the model. In order to facilitate insights regarding how regional metrics contribute into age prediction, we used a linear kernel with SVR, which assigns weights to features (i.e., regional values) reflecting their contributions. We used the SVR package with default settings implemented in Scikit-learn library [56] for Python [57].

We trained six SVR models to predict the age of participants, five with individual metrics (volume, surface area, thickness, FA, ADC) and one with combined metrics. An individual model using only a diffusion metric had 176 features corresponding to regional mean FA or ADC values. For individual models using only anatomical metrics, there were 68 features corresponding to regional mean volume, thickness, or surface area. Thus, the combined model using all modalities had 556 features. We did not use an external feature selection procedure, and the importance of features was determined using the feature weights as calculated by the SVR algorithm.

Each SVR model was trained using only the TDC sample to define a normative model of development. Prediction accuracy of the normative models was assessed using 10-fold cross-validation, repeated 1000 times in randomized order. In each fold, 10% of the data was kept for testing and the rest was used for training the SVR model. The ages of the participants in the test sample were then predicted using the trained model. This was repeated (10-fold) to use all participants once in the test sample, and the accuracy was calculated as Pearson correlation between predicted and actual age. Finally, the entire procedure was repeated 1000 times (i.e., 1000 times 10-folds), after randomly changing the order of participants, yielding 1000 Pearson *r* values. We reported the mean accuracy and 95% confidence interval using this distribution of *r* values.

We also demonstrated that the reported results are not model-dependent by repeating all analyses using two more regression models, namely Lasso and Bayesian regression. The use of Bayesian regression, by providing metrics on prediction uncertainty, also allowed us to incorporate into the model uncertainty induced by the availability of data across ages and variation across people in the training sample, to better capture individual differences [37, 38]. Results as reported in Additional file 1: Section S3 were highly similar to the original results reported below, demonstrating that our results are not model-dependent, nor driven by unaccounted uncertainty.

### Developmental deviation index

The normative age prediction model (SVR), trained using the TDC sample, was used to predict ages of participants with ASD, yielding their *brain age*. We calculated the difference between predicted age (brain age) and the chronological age for each participant with ASD, as an index of deviation from normative development, called *developmental deviation index* (DDI). When using a linear regression model, the regression towards the mean effect [58] results in younger ages being systematically overestimated and older ages being systematically underestimated. Therefore, the difference between brain age and chronological age is expected to be correlated with chronological age, obscuring real developmental deviation [59]. Thus, we adjusted the DDI for the regression towards the mean effect, by regressing out the chronological age from the DDI values [39]. For ease of interpretation, DDI was normalized to have a mean of 0 and standard deviation of 1.

### Statistical metrics

The correlation between age and neurobiological metrics (FA, ADC, surface area, volume, thickness) was calculated using Pearson correlation coefficient *r*. The statistical significance of the difference between the TDC and ASD groups, in terms of correlation between age and the neurobiological metrics, was assessed using Fisher *r* to *z* transformation. We only tested for regions whose age correlation was significant (*p* < 0.05) in both groups.

The correlation between the DDI and the ADOS severity was calculated using Spearman’s rank correlation coefficient due to the ordinal nature of ADOS severity scores. The effect size of difference in ADOS severity scores, for the same reason, was reported using common-language effect size [60]. The common-language effect size is the probability of having higher/lower severity in one of the groups, and is easy to interpret since it reports a probability value [61]. The Cohen’s *d* values were also reported for the sake of completeness, but they should be interpreted carefully, as ADOS severity scores are not distributed normally.

The false discovery rate (FDR) technique [62] was used for multiple comparisons correction. When studying correlations between age and individual metrics, FDR was used for each metric individually, correcting for multiple comparisons due to multiple brain regions.

The 95% confidence intervals (CI) for Pearson *r* values were calculated using Fisher *r* to *z* transformation. With the SVR model of the normative age prediction (using the TDC sample), we had 1000 *r* values (1000 times 10-fold cross validation); thus, we are able to estimate the standard error of the *z* distribution from those 1000 values. In other cases (e.g., testing accuracy with the ASD sample), the standard error was estimated based on sample size ($1/\sqrt{N-3}$).

In order to test the robustness of our findings against MRI motion and IQ differences, we fitted an ordinal regression model to predict ADOS severity scores using the DDI, MRI motion, and total IQ (Severity ~ DDI + Motion + IQ). We used the *ordinal* package [63] in R [64].

## Results

We first investigated developmental changes in anatomical and diffusion metrics of the brain, both in typical development and in ASD, in order to elucidate diverse effects of age on those metrics across different brain tissues and regions. We studied developmental alteration/similarity in ASD as compared with TDC. We then defined a normative model of multimodal brain development by fusing diverse age affects across brain regions, using a multivariate machine learning model. Finally, we studied how individual-level deviation from this normative model related to behavioral variation in ASD. Specifically, we defined distinct subgroups of ASD, each having different deviation from the normative model and compared them in terms of symptom severity values. We also demonstrated a dimensional relationship between individual-level deviation and disorder symptom severity.

### Patterns of regional brain maturation

**Fig. 1:**
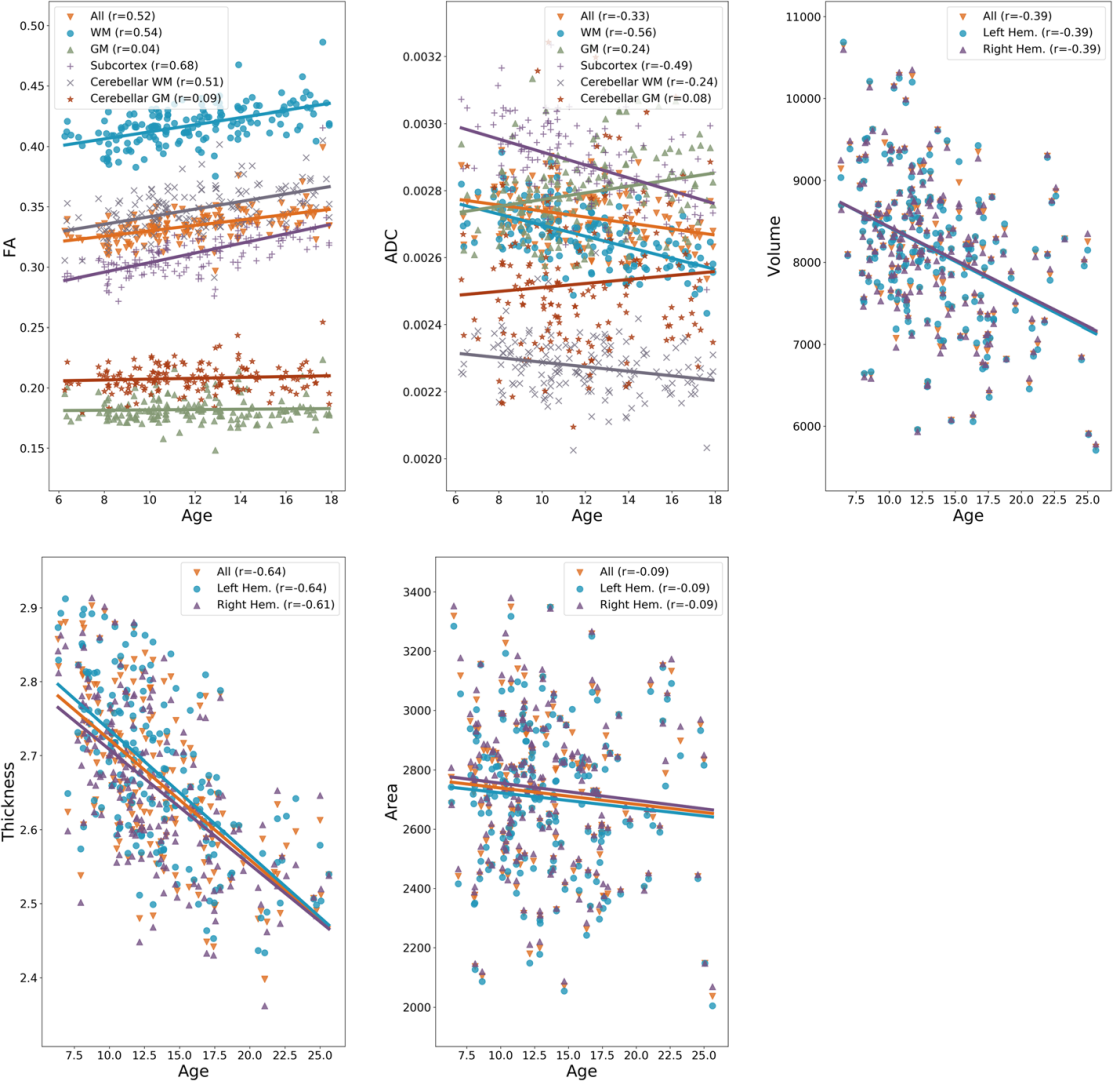
Global patterns of normative brain maturation for diffusion (FA, ADC) and anatomical (surface area, volume, thickness) metrics. Note that the brain parcellation used for anatomical metrics included only cortical GM. Heterogeneous effects of age on diffusion metrics are observed across tissue types. All anatomical metrics decline with age, with a similar trend in both hemispheres

**Fig. 2:**
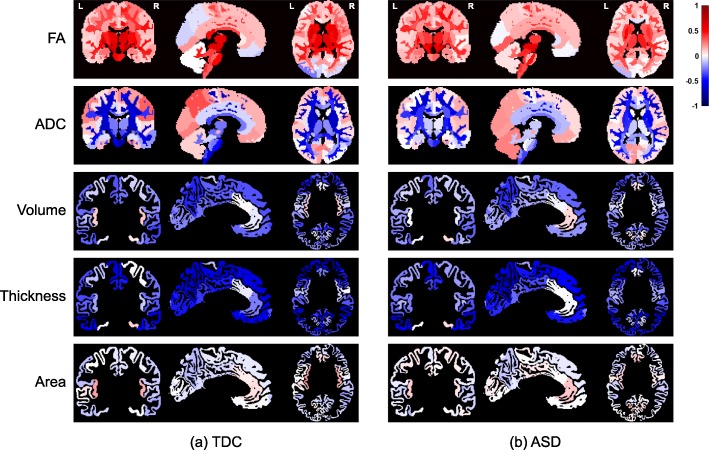
Regional patterns of brain maturation for diffusion (FA, ADC) and anatomical (surface area, volume, thickness) metrics, within **a** TDC and **b** ASD samples. Colors correspond to Pearson correlation (*r*) between age and anatomical/diffusion metrics. All regions are color-coded regardless of their *p* values to better visualize the overall age effects. Note that the brain parcellation used for anatomical metrics included only cortical GM

We studied how anatomical and diffusion metrics of the brain change with age within the TDC sample, to elucidate normative patterns of brain maturation. We observed divergent effects of age on diffusion metrics across tissue types, as seen in Fig. 1, with increase in certain tissue types and decrease or no change in others. The effects of age were more homogenous on anatomical metrics. The cortical volume and cortical thickness, on average, decreased in both hemispheres. The cortical surface area, on average, did not change with age in either of hemispheres. Details are given in Additional file 1: Section S2. The effects of maturation were heterogeneous across brain regions as illustrated in Fig. 2a, with correlation between age and all metrics showing variations across regions.

Overall patterns of maturation in the ASD sample were very similar to that of the TDC sample for all anatomical and diffusion metrics as shown in Fig. 2b and Additional file 2: Figure S1. In ASD, the absolute correlation values were slightly decreased with FA, ADC, and cortical thickness (see Additional file 3: Table S1 for details). Nevertheless, after multiple comparison correction, results remained significant only with ADC metric and only in the seven regions listed in Additional file 3: Table S1, suggesting an agreement between maturation patterns in TDC and in ASD.

### Multivariate-multimodal brain development

**Fig. 3:**
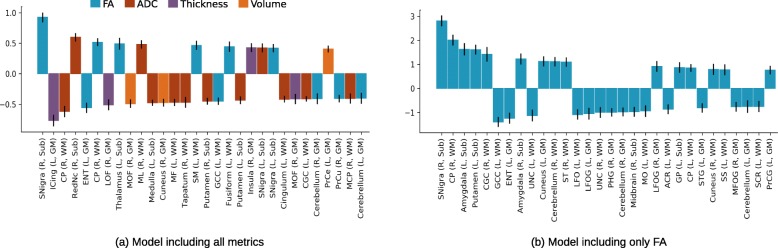
The weights of the brain regions in age prediction within the TDC sample, using **a** all five metrics (volume, surface area, thickness, FA, and ADC) and **b** only FA. The second model is included here as it is the only model with a significant correlation between the DDI and ASD symptom severity. Only top 30 features are visualized. Most features were related to FA and ADC metrics in the combined model. Higher absolute values indicate bigger contribution in the prediction. Note that the weights should not be compared between the two models, as the difference in magnitudes can be mostly explained by the number of features used in models. The abbreviations used: L, left; R, right; Sub, subcortical; GM, gray matter; WM, white matter; Snigra, substantia nigra; ICing, isthmus of cingulate; CP, cerebral peduncle; RedNc, red nucleus; ENT, entorhinal; LOF, lateral orbitofrontal; MOF, medial orbitofrontal; ML, medial lemniscus; MF, middle frontal; SM, supramarginal; GCC, genu of corpus callosum; CGC, cingulum (cingulate gyrus); PrCe, precentral; PrCu, precuneus; MCP, middle cerebellar peduncle; UNC, uncinate; ST, superior temporal; LFO, lateral fronto-orbital; LFOG, lateral fronto-orbital gyrus; PHG, parahippocampal gyrus; MO, middle occipital; ACR, anterior corona radiata; GP, globus pallidus; STG, superior temporal gyrus; SS, sagittal stratum; MFOG, middle fronto-orbital gyrus; SCR, superior corona radiata; PrCG, precentral gyrus

The SVR model using all metrics in combination achieved a high 10-fold cross-validation accuracy in predicting age (brain age) within the TDC sample (Pearson correlation between chronological and brain age, *r* = 0.88, CI = [0.87–0.89]). The feature weights that reflect contribution of regional metrics towards age prediction in the TDC sample are given in Fig. 3 for the top 30 features. As can be seen in the Fig. 3a, diffusion metrics (FA and ADC) were dominant in predicting age, representing 23 of the top 30 features. Similarly, diffusion metrics, in general, compared with anatomical metrics, achieved higher cross-validated prediction accuracy when normative models were trained using individual metrics (ADC: *r* = 0.87, CI = [0.85–0.88]; FA: *r* = 0.80, CI = [0.78–0.81]; thickness: *r* = 0.79, CI = [0.78–0.80]; volume: *r* = 0.66, CI = [0.64–0.68]; area: *r* = 0.22, CI = [0.16–0.27]).

The normative models achieved very high testing accuracy when tested with the ASD sample, both using all metrics in combination (Pearson *r* = 0.85, CI = [0.80–0.89]) and using individual metrics (ADC: *r* = 0.78, CI = [0.72–0.83]; FA: *r* = 0.76, CI = [0.69–0.82]; thickness: *r* = 0.79, CI = [0.74–0.84]; volume: *r* = 0.59, CI = [0.50–0.67]; area: *r* = 0.26, CI = [0.13–0.38]).

### Deviation from normative development and ASD severity

Among six SVR models (five individual models and one combined model), only one model, using the FA metric only, revealed significant correlation between the DDI and the symptom severity. This remained significant after multiple comparison correction for the six models. Below we report results using the FA model; others can be seen in Additional file 4: Table S2.

**Fig. 4:**
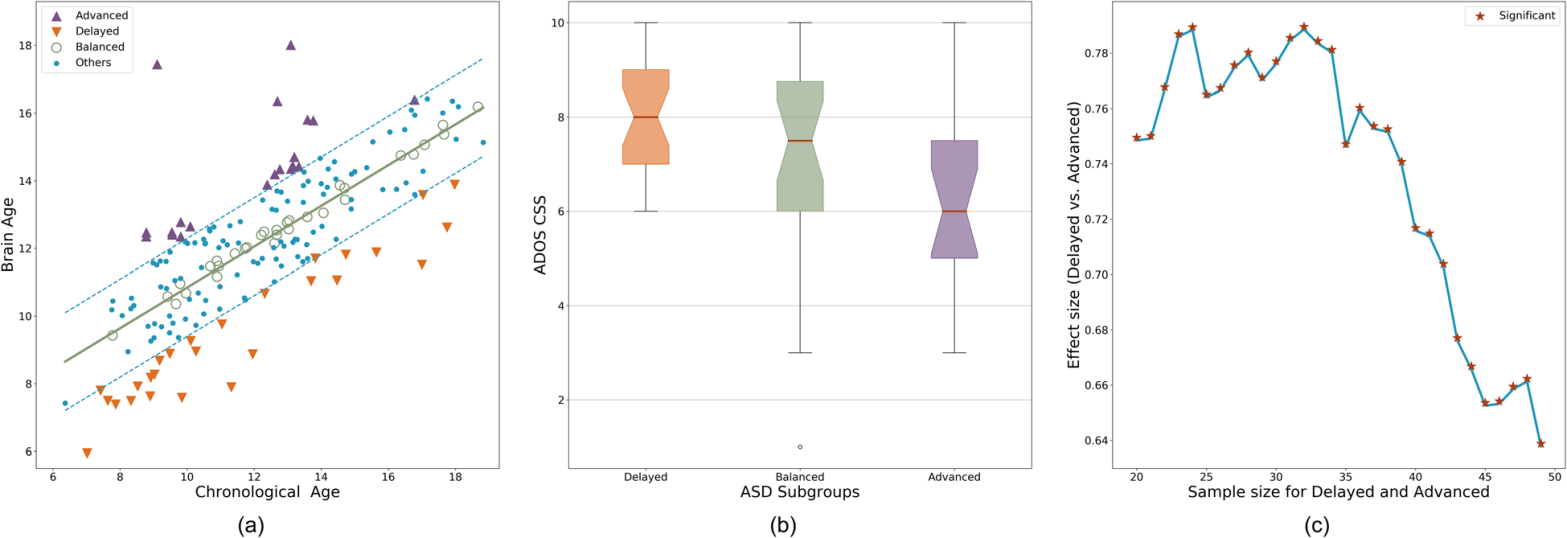
**a** Individuals with ASD were grouped into three subgroups based on the DDI values. Advanced group had higher brain age compared with chronological age (DDI > = 1). Delayed group had lower brain age compared with chronological age (DDI < = − 1). Balanced group had similar brain age and chronological age (− 0.2 < DDI < 0.2). **b** ASD severity values for the three subgroups. **c** The effect size of group difference between Advanced and Delayed groups. The effect size is reported as common-language effect size (i.e., probability of having higher severity in the Delayed group), which is an appropriate choice for ordinal severity values. The effect size was calculated for varying number of people in each group (adjusting DDI threshold accordingly) to demonstrate the robustness of group difference to the DDI threshold. Regardless of the sample sizes, the inter-group difference was always significant (*p* < 0.05)

A total of 176 participants with ASD had both diffusion imaging and ADOS calibrated severity score. In order to investigate the developmental heterogeneity in ASD, we defined three subgroups, as listed in Table 2, namely (1) Advanced group (*n* = 19) including participants with DDI > = 1, (2) Delayed group (*n* = 27) including participants with DDI < = − 1, and (3) Balanced group (*n* = 26) including participants with − 0.2 < DDI < 0.2. Note that DDI values were normalized to have a standard deviation of 1. The threshold for the Balanced group was chosen to have a sample size (~ 25) that is comparable to the other two groups. A notable hierarchy was observed among the three subgroups (Fig. 4) in terms of their symptom severity (Delayed > Balanced > Advanced; Kruskal-Wallis *H*-test statistic = 7.84, *p* = 0.0198) with a substantial effect size between the Delayed and Advanced subgroups (Cohen’s *d* = 1.01, common-language effect size = 0.74; Mann-Whitney test statistic = 380.5, *p* = 0.0051). The difference between the Delayed and Advanced subgroups was robust to the choice of the DDI thresholds as illustrated in Fig. 4c. There was no significant group difference between the two groups in terms of age (Cohen’s *d* = − 0.09, common-language effect size = 0.45; Mann-Whitney test statistic = 241.0, *p* = 0.5397) or IQ (Cohen’s *d* = − 0.24, common-language effect size = 0.44; Mann-Whitney test statistic = 225.0, *p* = 0.4887).

**Table 2:** Characteristics of ASD subgroups defined by DDI values

ASD subgroups	Age Mean (Std)	Total IQ Mean (Std)	ADOS CSS Mean (Std)
Delayed	11.53 (3.38)	95.52 (16.41)	8.00 (1.19)
Balanced	12.97 (2.68)	91.27 (24.62)	7.04 (2.08)
Advanced	11.80 (2.13)	99.32 (14.32)	6.47 (1.87)

**Fig. 5:**
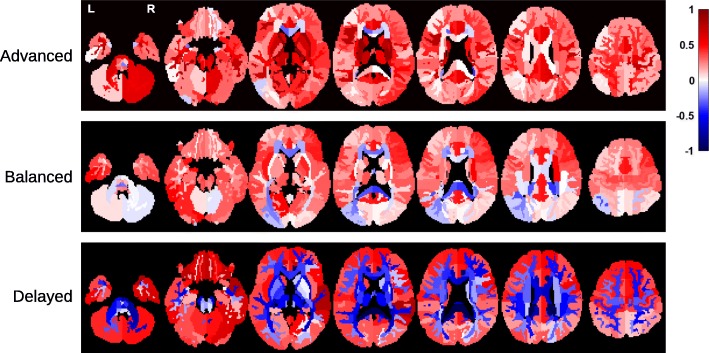
Neuroimaging (FA) differences between the TDC sample and the three subgroups of ASD, namely Advanced, Balanced, and Delayed. Colors correspond to effect size of group comparison (Cohen’s *d*), with positive values indicate higher FA values in ASD. All regions, regardless of *p* values, are visualized. The Advanced group, in average, had higher FA values compared with the TDC sample. The effect sizes become smaller across regions in the Balanced group. In the Delayed group, we see dominantly negative effect sizes. The hierarchy between the subgroups in terms of symptom severity (Delayed > Balanced > Advanced) is preserved (in the reversed direction) with FA values as well

Figure 5 illustrates neuroimaging (FA) differences between the TDC sample and the three subgroups of ASD. The regions with significant differences (after multiple comparison correction) are listed in Additional file 5: Table S3. With the Balanced group, no regional difference survived the multiple comparison correction. The sign of effect size (whether ASD is higher or lower in FA) in Fig. 5 agrees with the sign of DDI values of the subgroups (Advanced > Balanced > Delayed) in most regions; that is, the Advanced group had higher FA values compared with the TDC sample, whereas the Delayed group had lower values. The Balanced subgroup had lower effect sizes compared with other two subgroups. Please note that these results can be partly explained by the fact that the DDI values are indirectly computed from deviation of FA values with respect to the TDC sample; thus, higher FA values are expected for a subgroup with positive DDI values.

Beyond the categorical group differences, a dimensional relationship between brain maturation and behavior was also observed within the whole ASD sample. The DDI was significantly correlated with the disorder symptom severity (Spearman *r* = − 0.20, CI = [− 0.34 to − 0.05], *p* = 0.0076). In order to test the robustness of correlation between the DDI and symptom severity to motion during MRI acquisition and IQ, we fit an ordinal regression model to predict ADOS severity scores using the DDI, motion, and total IQ. The DDI had the most dominant contribution in the model (coefficient = − 0.32, *p* = 0.0166) followed by IQ (coefficient = − 0.01, *p* = 0.0264), and motion was not a significant predictor of severity (coefficient = 0.13, *p* = 0.6253). In other words, the correlation between the DDI and symptom severity remained significant when we consider MRI motion and IQ of participants.

### Sex differences

In our dataset, the TDC and ASD samples were not matched in sex ratio (see Table 1; *χ*^2^ = 5.49, *dof* = 1, *p* = 0.0191). Thus, in order to examine possible contribution of sex differences to the reported results, we compared the DDI values between males and females with ASD. This yielded no notable difference between sexes (Cohen’s *d* = 0.04, common-language effect size = 0.49; Mann-Whitney test statistic = 2322.0, *p* = 0.8619). We also reran all analyses including only males in both the TDC and ASD samples. The correlation between the DDI and symptom severity was almost identical to the original result above (Spearman *r* = − 0.20, CI = [− 0.35 to − 0.04], *p* = 0.0125).

## Discussion

In this work, we systematically studied developmental changes on anatomical (surface area, thickness, and volume) and diffusion (FA, ADC) metrics of the brain in normative development and in ASD. We demonstrated diverse age effects in different brain tissue types and regions, depicting a heterogeneous maturation pattern across the whole brain. By fusing these diverse age effects within a multivariate and multimodal machine learning model, we showed that anatomical and diffusion metrics were able to capture brain development with high accuracy. On average, brain maturation patterns, as captured by imaging metrics, were similar between TDC and ASD samples, suggesting preserved core developmental patterns in ASD. On the other hand, at the individual level, deviation from normative development, as computed by the DDI, was significantly correlated with the symptom severity in ASD, highlighting importance of individual-level analysis in disorder samples with heterogeneous presentations.

### Regional brain maturation

We reported regional patterns of brain maturation as quantified by correlations between age and several anatomical and diffusion metrics. Our results, showing increased FA and decreased ADC in WM and subcortical regions, agree with previous reports in normative samples [65, 66]. While it is difficult to pinpoint exact mechanisms (e.g., increasing myelination, increasing axonal density, or axonal pruning) yielding these results without detailed microstructural analyses [67], results are consistent with the expected brain connectivity maturation within the age range of our sample [68, 69]. Notably, both cortical volume and thickness decreased with age. In contrast, cortical surface area did not show a notable change with age; however, this may be due to our use of a linear correlation coefficient, since a few previous studies demonstrated that maturation in anatomical measures follow a cubic or quadratic trend in some areas across this age range [70–72].

We observed age-related differences between the TDC and ASD samples in FA, ADC, and cortical thickness, with small to moderate effect sizes (difference in *r* in the range of 0.13–0.39), although results did not survive multiple comparison correction in our sample, except for ADC in a few regions. Our results, in average, agree with previously reported age-related differences in diffusion [73–77] and anatomical [78–80] metrics in ASD. In our sample, the absolute values of correlation coefficients were decreased in ASD, supporting previous findings of flattened curves of maturation in ASD [73, 76, 79]. The lack of substantial (i.e., not significant after multiple comparison correction) group differences may suggest preserved developmental mechanisms in ASD at the group level; however, this needs further investigations, possibly including nonlinear metrics of correlation. Similar findings, with ASD-related alterations being obscured at the group level while present at the individual level, were reported previously, using a normative modeling approach [81].

### Multivariate brain maturation

We observed a diverse effect of maturation in the brain, with effect of age varying across brain tissues and regions, highlighting the need for using advanced multivariate techniques to better capture inter-regional interactions. Our results with multivariate prediction models (SVR and two other regression models) clearly demonstrated that imaging-based anatomical and diffusion metrics of the brain can effectively capture developmental changes, as suggested by high age prediction accuracy. The tissue microstructure and possibly connectivity-related diffusion measures (ADC and FA) had the highest prediction accuracy, followed by cortical thickness, volume, and surface area. Higher predictive power of diffusion metrics may be due to the age range of our sample, reflecting a specific phase of development when WM matures most. A multimodal prediction model, combining all modalities had the highest prediction accuracy. This was despite the substantial decrease in the number of participants having all metrics and increase in dimensionality, which is known to hurt the prediction performance [82]. These results highlight the complementary nature of anatomical and diffusion metrics, with a relatively higher contribution of diffusion metrics.

In the present study, among the six multivariate models, only one model trained with a single diffusion metric (FA) had a notable correlation between the DDI and symptom severity. On the contrary, the normative model combining all modalities and metrics performed best in age prediction. These two findings, taken together, may suggest that the neuroimaging metrics capture complementary aspects of brain maturation, and ASD-related alterations are mostly linked to a specific aspect that is captured by the FA metric, possibly associated with tissue microstructure and brain connectivity. Such a hypothesis requires further evaluations with replication studies; nevertheless, it supports the previous reports suggesting links between behavioral profiles in ASD and altered brain diffusion characteristics and connectivity [28, 83, 84]. Indeed, many studies have reported connectivity-related alterations in ASD, suggesting that ASD is a disorder of brain connectivity [85, 86]. Our results expand this conceptualization by suggesting that ASD severity might be related to atypical development in white matter tissue characteristics.

The lack of correlation between symptom severity and the DDI defined by anatomical metrics in our sample should not be taken as a disagreement with previous studies suggesting links between anatomical metrics and behavior [13, 87–89]. It is essential to note that we did not test whether symptom severity correlated with the diffusion and anatomical metrics themselves, or whether the brain measures accurately classified diagnosis. Rather, we tested whether their deviation from the typical development correlated with symptom severity. Given the smaller relationship between age and anatomical measures, particularly surface area, compared with diffusion metrics, it is perhaps unsurprising that the deviation from normative development in these measures does not contain sufficient variation to predict severity. A significant correlation between severity of repetitive behaviors and deviation from normative brain anatomy was reported recently, using another normative modeling approach [81], where each voxel of the brain was analyzed independently. It is possible that aggregating deviations from all voxels and regions into a single score, as done in our approach, may have caused a loss of variation necessary to detect anatomy-behavior relationship. This hypothesis can be tested in future studies by directly comparing different normative modeling approaches within the same sample.

### Developmental heterogeneity in ASD

Although regional and global developmental patterns of anatomical and diffusion metrics, on average, were similar between the TDC and ASD samples, our individual-level analysis within the ASD sample revealed significant links between deviation from normative development and disorder symptom severity. We investigated developmental heterogeneity in ASD, by using the DDI to stratify the ASD sample into three developmental subgroups, namely Advanced, Balanced, and Delayed subgroups. We demonstrated a significant hierarchy among the subgroups in terms of mean symptom severity (Advanced < Balanced < Delayed). Going beyond these categorical differences, we also observed a significant correlation between symptom severity and the DDI in the whole ASD sample as well. Our methods and similar stratification techniques can be easily extended to other psychiatric conditions [90] and to other data modalities.

Our results portray a heterogonous picture of ASD, as also suggested by previous studies [91, 92]. Two subgroups of ASD, the Advanced and Delayed subgroups, had neuroimaging differences from the TDC sample in the reversed directions. Within the Advanced group, ASD was associated with higher regional FA values compared with typically development, whereas, lower FA values were present in the Delayed group. Both groups had FA differences in the brain regions previously reported in ASD, such as internal capsule [93–96], external capsule [97, 98], and inferior temporal gyrus [99] in the Advanced group, corpus callosum [95, 97, 100–103], cerebellar peduncle [95, 97, 104], and inferior fronto-occipital fasciculus [98, 100] in the Delayed group, and cingulum [100, 105–107] in both groups. Notably, the regions that showed significant differences were not same for the subgroups. Such a picture supports the idea of *equifinality* in psychiatric disorders [41] that suggests a single behavioral outcome may be linked to distinct underlying mechanisms in different subgroups or even in each individual.

The Advanced group, in average, had less symptom severity not only compared with the Delayed group, but also compared with the Balanced group, which had typical brain age (i.e., close to chronological age). In other words, having advanced brain maturation, which may be considered to be a type of “deviation,” is associated with positive outcomes. Previous studies have reported increased cognitive performance for individuals with high brain age, in normative samples [39, 108]. Here we found higher brain age is related to reduced ASD severity. It seems that advanced brain maturation begets positive attributes both in normative and disorder samples. Notably, the Advanced and Delayed subgroups did not differ significantly in terms of IQ scores, and the negative correlation between symptom severity and the DDI remained significant after correcting for IQ differences. Thus, if we wish to speculate a parallel between reduced symptom severity and increased cognitive performance, we should consider cognitive domains that are associated with ASD symptomatology, such as social cognition, executive functioning, and theory of mind [109–114], rather than cognitive domains assessed by standardized IQ measures (i.e., verbal and nonverbal reasoning).

## Limitations

Interpretation of the reported results should be made in the light of several limitations of the work. First of all, brain maturation is a dynamic process with different trajectories in different age ranges [20]. Our results only reflect the links between age and certain anatomical and diffusion metrics as observed within the age range of the study sample (6–25 years, mean = 13.0, Std = 3.8). A comprehensive portrait of the brain development in ASD requires datasets that include a wide range of ages, from infancy to late adulthood. Moreover, our work characterizes brain development only through MRI-based metrics, and their overlap with the underlying biological processes is not well described [115].

We used linear models in order to facilitate better interpretations. More sophisticated yet complicated models could also have been used to possibly have better predictive power, for example, to have higher prediction accuracy in age prediction [116]. Nevertheless, using a simple linear predictor, we were able to achieve a substantial cross-validated accuracy, which effectively demonstrates the capability of our model in capturing the brain maturation patterns.

We performed a cross-sectional analysis to study developmental effects in ASD. More reliable inferences about brain development require longitudinal studies [117] that have the statistical capabilities to better characterize developmental trajectories and to better capture within- and between-person variations [118].

The brain age approach used in this study defines a normative model to predict age from neuroimaging metrics, which is the opposite of other normative modeling approaches that predict neuroimaging metrics from age [38]. The direction of prediction used in brain age approach explicitly defines a normative age model but not a normative model of neuroimaging metrics, which is available only implicitly (e.g., weights of SVR). Although it is always possible to invert the linear mapping between variables to facilitate interpretations on regional neuroimaging metrics, one may also use other normative modeling approaches to achieve this directly [37, 38].

Our results suggested that the DDI derived from only one neuroimaging metric (FA) showed a correlation with symptom severity. Such specific findings necessitate replication studies using other large datasets to demonstrate how these results generalize. Without such replication studies, it is not possible to exclusively attribute ASD-related neuroanatomical alterations to variations in a single diffusion metric.

Based on the reported moderate effect sizes, replication studies using possibly bigger samples are needed for this work to be reliably translated into any clinical decision making or treatment strategies.

## Future directions

ASD is a complex psychiatric condition characterized by diverse impairments in multiple domains of functioning [1] and presence of numerous comorbidities including deficits in cognitive function and language ability [119, 120], as well as co-occurring psychiatric and neurological conditions [121]. This complex clinical presentation of the disorder makes it a challenge to quantify the symptom severity using a single measure. Although we used one of the gold-standard measures of symptom severity (ADOS calibrated severity score), a detailed characterization of developmental heterogeneity necessitates studying links between brain development and symptom severity along multiple dimensions. In future work, we will study these multiple dimensions of developmental heterogeneity using our probabilistic data fusion techniques [122].

Our results indicated a notable contribution of diffusion metrics in explaining observed behavioral heterogeneity across development. It seems intuitive to conclude from these results that brain connectivity is a key factor in understanding neurobiological substrates of ASD, which is also supported by previous studies [85, 86]. Nevertheless, diffusion metrics used in this study only characterize the tissue microstructure, but not directly the connectivity between regions. Thus, future studies using direct measures of brain connectivity derived from connectomes [123] (e.g., connectivity strength between regions) are necessary to better understand neurobiological underpinnings of the disorder. Our methods introduced in this study can be easily used in such future studies of brain connectivity.

In future studies, our findings can be extended through use of longitudinal datasets in order to identify diverse trajectories of the DDI across ages. Maturation is a dynamic process; it is expected that an individual with ASD would have differential deviation from the normative brain maturation at different stages of development [20]. Investigations on the DDI dynamics across ages may provide further insights into to our understanding of the individualized pathways of the disorder, especially in relation to different treatment and intervention strategies.

## Supplementary information

**Additional file 1.** Supplementary notes

**Additional file 2: Figure S1.** Global patterns of brain maturation for diffusion (FA, ADC) and anatomical (surface area, volume, thickness) metrics in ASD. **Figure S2.** Results using Lasso regression. **Figure S3.** Results using Bayesian regression. **Figure S4.** Results using Bayesian regression, with uncertainty is explicitly modeled

**Additional file 3: Table S1.** Regional brain maturation differences between TDC and ASD samples.

**Additional file 4: Table S2.** Relationship between DDI and ASD Severity.

**Additional file 5: Table S3.** Neuroimaging (FA) differences between the TDC sample and the two subgroups of ASD.

## Data Availability

All data generated and/or analyzed, and the source code of all computer programs used during this study are available from the corresponding author on reasonable request.

## Abbreviations

ADC: Apparent diffusion coefficient
ADOS: Autism diagnostic observation schedule
ASD: Autism spectrum disorder
CAR: Center for autism research
CHOP: Children’s hospital of Philadelphia
CI: Confidence intervals
CSS: Calibrated severity score
DDI: Developmental deviation index
dMRI: Diffusion magnetic resonance imaging
FA: Fractional anisotropy
FDR: False discovery rate
IRB: Institutional review board
MRI: Magnetic resonance imaging
RRB: Restricted and repetitive behaviors
SA: Social affect
SVR: Support vector regression
TDC: Typically developing children
