# Supplementary material for: Deviation from normative brain development is associated with symptom severity in autism spectrum disorder
Source: Mol Autism. 2019 Dec 11;10:46. doi: 10.1186/s13229-019-0301-5 (PMC6907209; doi:10.1186/s13229-019-0301-5)
Supplement: Supplementary file 1 — Additional file 1. Supplementary notes [file 13229_2019_301_MOESM1_ESM.docx]

**Deviation from Normative Brain Development is Associated with Symptom Severity in Autism Spectrum Disorder**

Birkan Tunç, PhD^1,2,3,4^; Lisa D. Yankowitz^1,5^​, MA; Drew Parker, BSc^6^; Jacob A. Alappatt, BSc^6^; Juhi Pandey, PhD^1,3^; Robert T. Schultz, PhD^1,3,7^; Ragini Verma, PhD^6^

^1^ Center for Autism Research, The Children’s Hospital of Philadelphia, Philadelphia, PA 19104, USA.

^2^ Department of Biomedical and Health Informatics, The Children’s Hospital of Philadelphia, Philadelphia, PA 19104, USA.

^3^ Department of Psychiatry, University of Pennsylvania, Philadelphia, PA 19104, USA.

^4^ Center for Biomedical Image Computing and Analytics, Department of Radiology, University of Pennsylvania, Philadelphia, PA 19104, USA.

^5^ Department of Psychology, University of Pennsylvania, Philadelphia, PA 19104, USA.

^6^ DiCIPHR (Diffusion and Connectomics in Precision Healthcare Research) Lab, Department of Radiology, University of Pennsylvania, Philadelphia, PA 19104, USA.

^7^ Department of Pediatrics, University of Pennsylvania, Philadelphia, PA 19104, USA.

**SUPPLEMENTARY NOTES**

**S1. Image Processing and QA**

With T1-weighted anatomical images, brain extractions were visually inspected, and manually edited with ITK-SNAP [1] if cortex was removed by the automated extraction. Segmentations were visually inspected (blind to all subject characteristics) and manually edited using Freeview to correct segmentation errors. Final segmentations were visually inspected and excluded if motion artifacts clearly impacted segmentation quality, if image quality was very poor, or if a superior image was available in the case of subjects who had multiple data points available.

The QA was conducted to detect artifacts and outliers (including motion effects). Every volume of each scan was inspected visually. If the single b=0 image was found to be artifact-free and fewer than three diffusion weighted images were flagged as containing artifacts, the volume was kept, with any weighted volumes with artifact removed. Otherwise, the scan was flagged as a QA failure and that participant’s data was not included in the sample. This was followed by DWI de-noising using Slicer [2] and brain extraction using FSL [3]. Diffusion tensors were fitted to the DWI data using multivariate linear fitting [4] by in-house software. The DWI data were spatially normalized [5] to the Eve Atlas from the Johns Hopkins University School of Medicine with 176 white matter and gray matter ROIs [6]. We calculated average FA and ADC for each region.

Several participants had multiple sessions (*e.g.*, two DWI acquisitions). We used only one session per participant, selected based on QA results and so as to maximize the number of participants with both modalities in the same session.

**S2. Regional Brain Maturation**

FA, on average, increased with age globally (Pearson *r* = 0.52, *p* = 1.7x10^-12^), in cerebral white matter (WM) (*r* = 0.54, *p* = 8.8x10^-14^), in subcortical areas (*r* = 0.68, *p* = 6.3x10^-23^), and in cerebellar WM (*r* = 0.51, *p* = 3.2x10^-12^), whereas it did not change with age in cerebral gray matter (GM) (*r* = 0.04, *p* = 0.63) and cerebellar GM (*r* = 0.09, *p* = 0.24). ADC, on average, decreased with age globally (Pearson *r* = -0.33, *p* = 1.5x10^-5^), in cerebral WM (*r* = -0.56, *p* = 6.8x10^-15^), in subcortical areas (*r* = -0.49, *p* = 4.7x10^-11^) and in cerebellar WM (*r* = -0.24, *p* = 0.0018), whereas it increased in cerebral GM (*r* = 0.24, *p* = 0.0022) and did not change in cerebellar GM (*r* = 0.08, *p* = 0.29).

The cortical volume, on average, decreased both globally (Pearson *r* = -0.39, *p* = 1.5x10^-8^) and in each hemisphere (Left: *r* = -0.39, *p* = 1.0x10^-8^; Right: *r* = -0.39, *p* = 2.5x10^-8^). The cortical thickness, on average, showed even bigger decline with age, globally (*r* = -0.64, *p* = 1.4x10^-23^) and in both hemispheres (Left: *r* = -0.64, *p* = 3.5x10^-24^; Right: *r* = -0.61, *p* = 2.3x10^-21^). Notably, the cortical surface area, on average, did not change with age, and this was true globally (*r* = -0.09, *p* = 0.21) and for each hemisphere separately (Left: *r* = -0.09, *p* = 0.23; Right: *r* = -0.09, *p* = 0.19).

**S3. Using Other Regression Models**

Using SVR, we were able to predict chronological age of participants with high accuracy, and the DDI derived from SVR significantly correlated with ADOS severity. In order to demonstrate that the results are not dependent on the model choice, we repeated the analyses using two other commonly used regression models, namely Lasso [7] and Bayesian regression [8]. The use of Bayesian regression, by providing metrics on prediction uncertainty, also allowed us to incorporate into the model uncertainty induced by availability of data across ages and variation across people in the training sample. The results with the both models were very similar to the original results reported in the main text. Below we report them in an order that parallels the main text.

Lasso Regression: We used Lasso implementation of the Scikit-learn library [9] for Python [10]. We adjusted the sparsity level ($\alpha$ parameter) to get the best training accuracy, which was 0.01. The model using all metrics in combination achieved a high 10-fold cross-validation accuracy in predicting age within the TDC sample (Pearson correlation between chronological and brain age, r = 0.90, CI = [0.88 – 0.91]). The cross-validated prediction accuracy for the normative models trained using individual metrics were as follows: ADC: r = 0.83, CI = [0.81 – 0.85]; FA: r = 0.84, CI = [0.82 – 0.85]; Thickness: r = 0.79, CI = [0.77 – 0.80]; Volume: r = 0.64, CI = [0.61 – 0.67]; Area: r = 0.17, CI = [0.12 – 0.22]).

The test accuracy of the normative models within the ASD sample were as follows: All: r = 0.86, CI = [0.81 – 0.90]; ADC: r = 0.79, CI = [0.73 – 0.84]; FA: r = 0.77, CI = [0.70 – 0.82]; Thickness: r = 0.77, CI = [0.71 – 0.82]; Volume: r = 0.60, CI = [0.50 – 0.68]; Area: r = 0.26, CI = [0.13 – 0.38]).

Among six SVR models (five individual models and one combined model), only one model, using the FA metric only, revealed significant correlation between the DDI and the symptom severity. A notable hierarchy was observed among the three subgroups in terms of their symptom severity (Delayed > Balanced > Advanced; Kruskal-Wallis H-test statistic = 8.57, p = 0.0138) with a substantial effect size between the Delayed and Advanced subgroups (Cohen’s d = 1.04, common-language effect size = 0.76; Mann-Whitney test statistic = 382.0, p = 0.0028). The results with ASD subgroups are also given in Supplementary Figure S2. Finally, the DDI was significantly correlated with the disorder symptom severity (Spearman r = -0.15, CI = [-0.29 – -0.002], p = 0.0466).

Bayesian Regression: We used Bayesian ridge regression implementation of the Scikit-learn library [9] for Python [10], with its all default parameters. Similar to Lasso model, in Bayesian ridge regression, the coefficient weights are slightly shifted toward zeros, which stabilizes them. The prediction accuracy within the TDC sample for the model using all metrics in combination was again high (Pearson correlation between chronological and brain age, r = 0.88, CI = [0.86 – 0.89]). The prediction accuracy for the normative models trained using individual metrics were as follows: ADC: r = 0.86, CI = [0.85 – 0.87]; FA: r = 0.81, CI = [0.80 – 0.82]; Thickness: r = 0.79, CI = [0.78 – 0.80]; Volume: r = 0.67, CI = [0.65 – 0.69]; Area: r = 0.21, CI = [0.18 – 0.24]).

The test accuracy of the normative models within the ASD sample were as follows: All: r = 0.86, CI = [0.80 – 0.89]; ADC: r = 0.80, CI = [0.74 – 0.85]; FA: r = 0.76, CI = [0.70 – 0.82]; Thickness: r = 0.79, CI = [0.73 – 0.83]; Volume: r = 0.60, CI = [0.51 – 0.68]; Area: r = 0.25, CI = [0.12 – 0.37]).

Only one model, using the FA metric, revealed significant correlation between the DDI and the symptom severity. The hierarchy among the three subgroups in terms of their symptom severity was preserved (Delayed > Balanced > Advanced; Kruskal-Wallis H-test statistic = 7.39, p = 0.0249) with a large effect size between the Delayed and Advanced subgroups (Cohen’s d = 0.91, common-language effect size = 0.74; Mann-Whitney test statistic = 407.0, p = 0.0046). The results with ASD subgroups are also given in Supplementary Figure S3. Finally, the DDI was significantly correlated with the disorder symptom severity (Spearman r = -0.15, CI = [-0.29 – -0.001], p = 0.0485).

Bayesian Regression with Uncertainty: We used the same Bayesian ridge regression implementation again with its default parameters. The Bayesian regression, in addition to predicted age for each participant, also provides an uncertainty value (variance) for the prediction. It is thus possible to incorporate into the model uncertainty induced by availability of data across ages. Another source of variation (*i.e.*, variation across people in the training sample) can also be modeled using the variance learned from the training sample. Thus, the final DDI value for the participant $i$ can be calculated as $DDI_{i}=\frac{\hat{y}_{i}-y_{i}}{\sqrt{\sigma_{i}^{2}+\sigma_{n}^{2}}}$ , where $\hat{y}_{i}$ is the predicted age, $y_{i}$ is the chronological age, $\sigma_{i}^{2}$ is the predictive variance (returned by Bayesian regression) and $\sigma_{n}^{2}$ is computed as the variance of residuals ($\hat{y}-y$) in the training sample. As in the main text, we also correct this DDI value for the regression to mean effect, by covariating out the chronological age.

Using new DDI values, only one model, using the FA metric, revealed significant correlation between the DDI and the symptom severity. The hierarchy among the three subgroups in terms of their symptom severity was preserved (Delayed > Balanced > Advanced; Kruskal-Wallis H-test statistic = 6.57, p = 0.0374) with a moderate effect size between the Delayed and Advanced subgroups (Cohen’s d = 0.68, common-language effect size = 0.71; Mann-Whitney test statistic = 498.0, p = 0.0081). The results with ASD subgroups are also given in Supplementary Figure S4. Finally, the DDI was significantly correlated with the disorder symptom severity (Spearman r = -0.15, CI = [-0.29 – -0.001], p = 0.0453).

**REFERENCES**

[1] P. A. Yushkevich *et al.*, “User-guided 3D active contour segmentation of anatomical structures: significantly improved efficiency and reliability,” *Neuroimage*, vol. 31, no. 3, pp. 1116–1128, Jul. 2006.

[2] S. Aja-Fernandez, M. Niethammer, M. Kubicki, M. E. Shenton, and C.-F. Westin, “Restoration of DWI data using a Rician LMMSE estimator.,” *IEEE Trans. Med. Imaging*, vol. 27, no. 10, pp. 1389–403, Oct. 2008.

[3] S. M. Smith, “Fast robust automated brain extraction,” *Hum. Brain Mapp.*, vol. 17, no. 3, pp. 143–155, Nov. 2002.

[4] C.-F. Westin, S. E. Maier, H. Mamata, A. Nabavi, F. A. Jolesz, and R. Kikinis, “Processing and visualization for diffusion tensor MRI.,” *Med. Image Anal.*, vol. 6, no. 2, pp. 93–108, Jun. 2002.

[5] Y. Ou, A. Sotiras, N. Paragios, and C. Davatzikos, “DRAMMS: Deformable registration via attribute matching and mutual-saliency weighting,” *Med. Image Anal.*, vol. 15, no. 4, pp. 622–639, 2011.

[6] K. Oishi *et al.*, “Atlas-based whole brain white matter analysis using large deformation diffeomorphic metric mapping: application to normal elderly and Alzheimer’s disease participants.,” *Neuroimage*, vol. 46, no. 2, pp. 486–99, Jun. 2009.

[7] R. Tibshirani, “Regression Shrinkage and Selection via the Lasso,” *J. R. Stat. Soc. B*, vol. 58, no. 1, pp. 267–288, 1996.

[8] J. Kruschke, *Doing Bayesian Data Analysis : a Tutorial Introduction with R.*, 2nd ed. Academic Press, 2014.

[9] F. Pedregosa *et al.*, “Scikit-learn: Machine Learning in Python,” *J. Mach. Learn. Res.*, vol. 12, pp. 2825–2830, 2011.

[10] G. Rossum, “Python Reference Manual,” CWI (Centre for Mathematics and Computer Science), Amsterdam, 1995.
