# Supplementary material for: Deviation from normative brain development is associated with symptom severity in autism spectrum disorder
Source: Mol Autism. 2019 Dec 11;10:46. doi: 10.1186/s13229-019-0301-5 (PMC6907209; doi:10.1186/s13229-019-0301-5)
Supplement: Supplementary file 2 — Additional file 2: Figure S1. Global patterns of brain maturation for diffusion (FA, ADC) and anatomical (surface area, volume, thickness) metrics in ASD. Figure S2. Results using Lasso regression. Figure S3. Results using Bayesian regression. Figure S4. Results using Bayesian regression, with uncertainty is explicitly modeled [file 13229_2019_301_MOESM2_ESM.docx]

**Deviation from Normative Brain Development is Associated with Symptom Severity in Autism Spectrum Disorder**

Birkan Tunç, PhD^1,2,3,4^; Lisa D. Yankowitz^1,5^​, MA; Drew Parker, BSc^6^; Jacob A. Alappatt, BSc^6^; Juhi Pandey, PhD^1,3^; Robert T. Schultz, PhD^1,3,7^; Ragini Verma, PhD^6^

^1^ Center for Autism Research, The Children’s Hospital of Philadelphia, Philadelphia, PA 19104, USA.

^2^ Department of Biomedical and Health Informatics, The Children’s Hospital of Philadelphia, Philadelphia, PA 19104, USA.

^3^ Department of Psychiatry, University of Pennsylvania, Philadelphia, PA 19104, USA.

^4^ Center for Biomedical Image Computing and Analytics, Department of Radiology, University of Pennsylvania, Philadelphia, PA 19104, USA.

^5^ Department of Psychology, University of Pennsylvania, Philadelphia, PA 19104, USA.

^6^ DiCIPHR (Diffusion and Connectomics in Precision Healthcare Research) Lab, Department of Radiology, University of Pennsylvania, Philadelphia, PA 19104, USA.

^7^ Department of Pediatrics, University of Pennsylvania, Philadelphia, PA 19104, USA.

**SUPPLEMENTARY FIGURES**


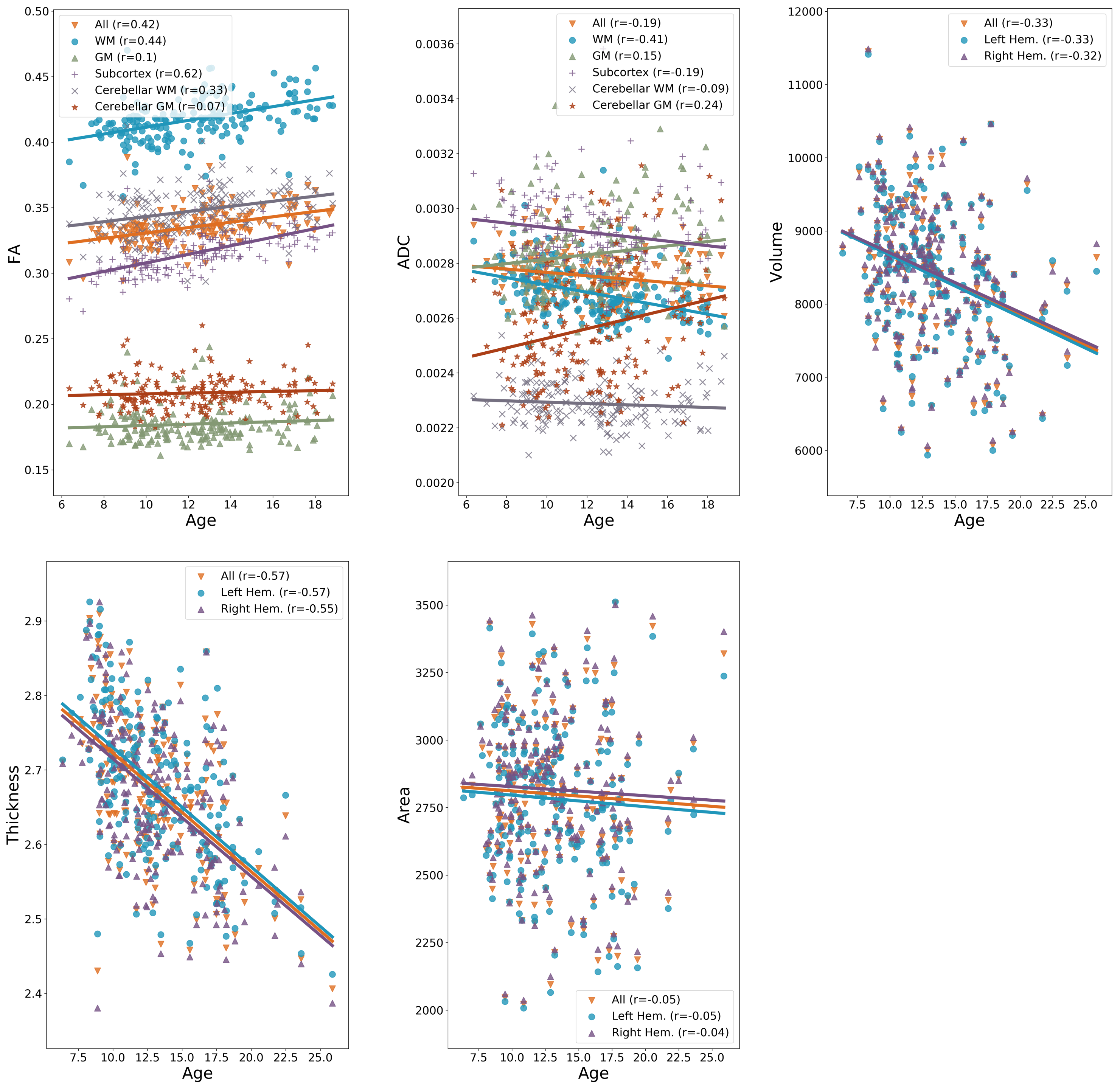


Figure S1. Global patterns of brain maturation for diffusion (FA, ADC) and anatomical (surface area, volume, thickness) metrics in ASD. Note that the brain parcellation used for anatomical metrics included only cortical GM. Heterogeneous effects of age on diffusion metrics are observed across tissue types. All anatomical metrics decline with age, with a similar trend in both hemispheres.

| 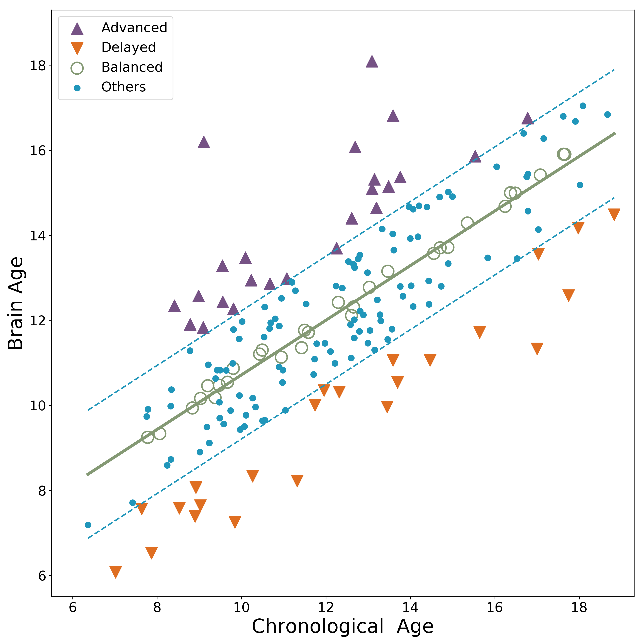  (a) | 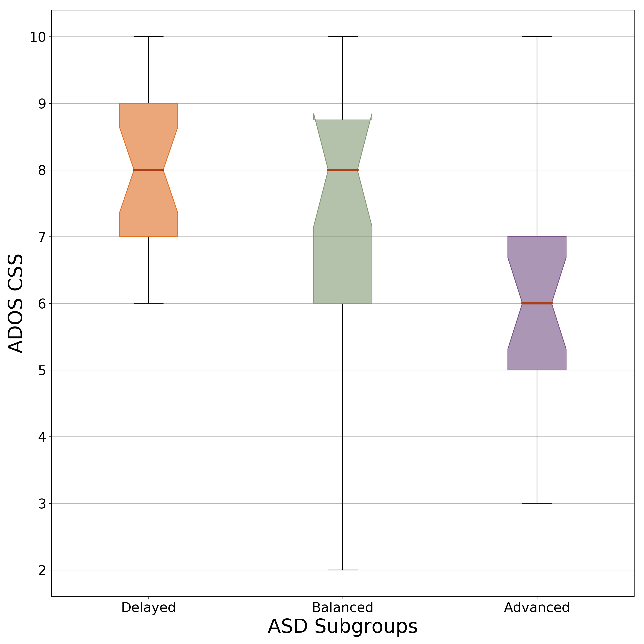  (b) |
| --- | --- |
| 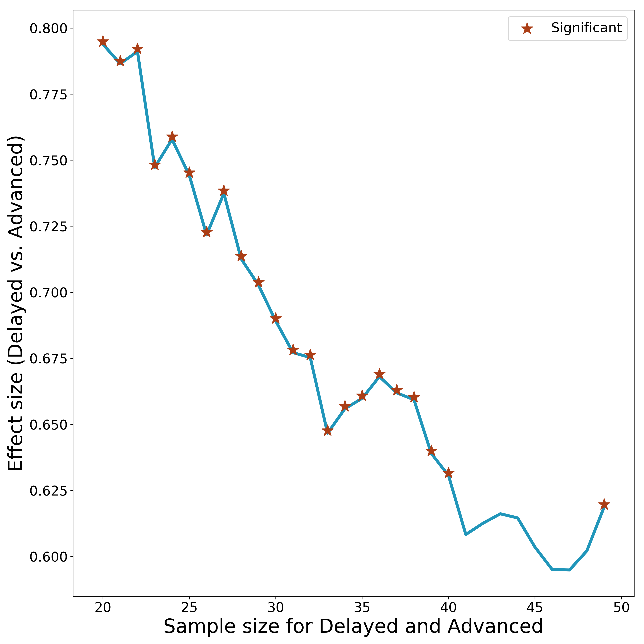  (c) | |

Figure S2. Results using Lasso regression. (a) Individuals with ASD were grouped into three subgroups based on the DDI values. Advanced group had higher brain age compared to chronological age (DDI >= 1). Delayed group had lower brain age compared to chronological age (DDI <= -1). Balanced group had similar brain age and chronological age ( -0.2 < DDI < 0.2). (b) ASD severity values for the three groups. (c) The effect size of group difference between Advanced and Delayed groups. The effect size is reported as common-language effect size (i.e., probability of having higher severity in the Delayed group), which is an appropriate choice for ordinal severity values. The effect size was calculated for varying number of people in each group (adjusting DDI threshold accordingly) to demonstrate the robustness of group difference to the DDI threshold. Regardless of the sample sizes, the inter-group difference was (almost) always significant (p < 0.05).

| 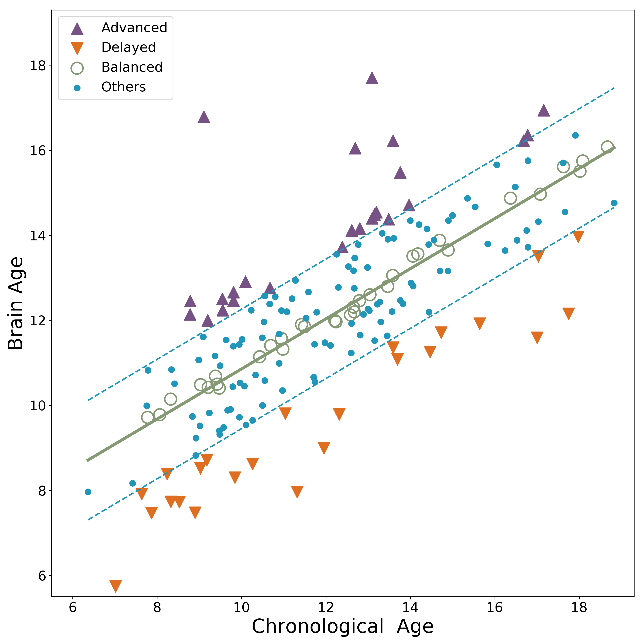  (a) | 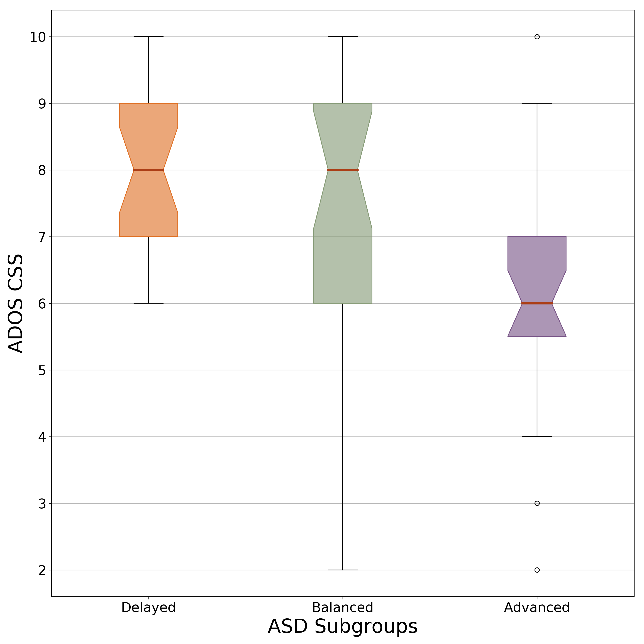  (b) |
| --- | --- |
| 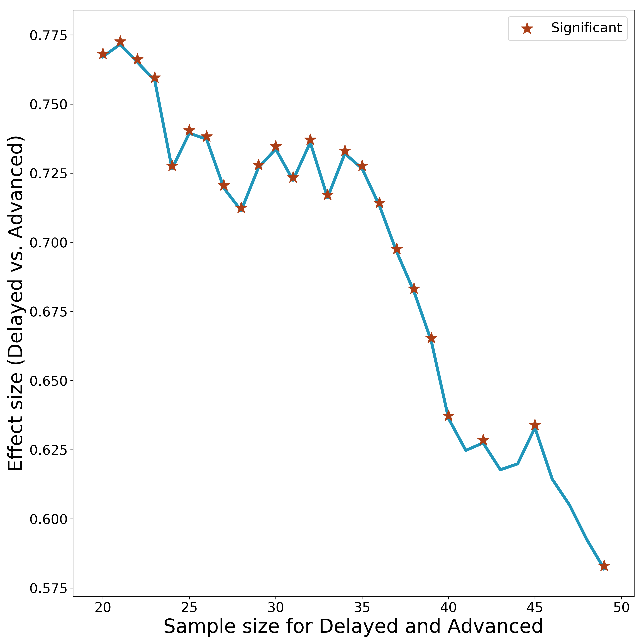  (c) | |

Figure S3. Results using Bayesian regression. (a) Individuals with ASD were grouped into three subgroups based on the DDI values. Advanced group had higher brain age compared to chronological age (DDI >= 1). Delayed group had lower brain age compared to chronological age (DDI <= -1). Balanced group had similar brain age and chronological age ( -0.2 < DDI < 0.2). (b) ASD severity values for the three groups. (c) The effect size of group difference between Advanced and Delayed groups. The effect size is reported as common-language effect size (i.e., probability of having higher severity in the Delayed group), which is an appropriate choice for ordinal severity values. The effect size was calculated for varying number of people in each group (adjusting DDI threshold accordingly) to demonstrate the robustness of group difference to the DDI threshold. Regardless of the sample sizes, the inter-group difference was (almost) always significant (p < 0.05).

| 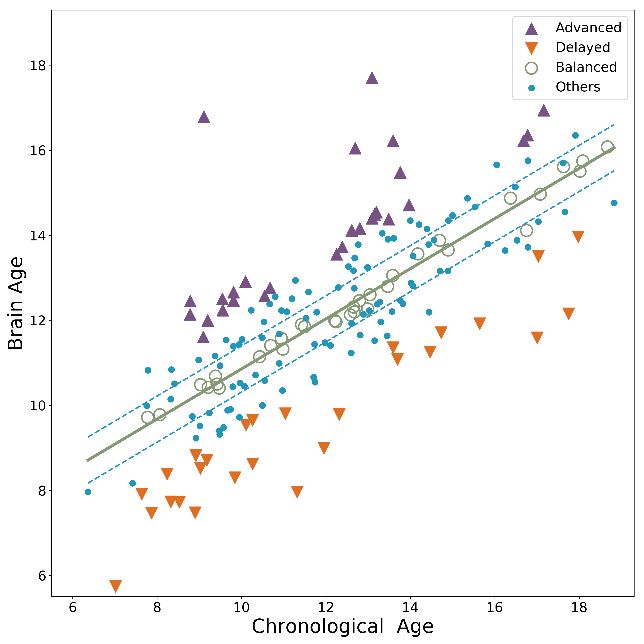  (a) | 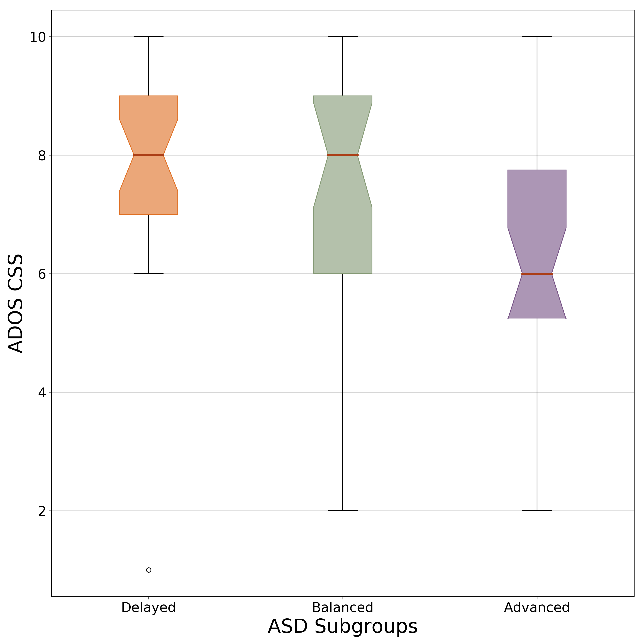  (b) |
| --- | --- |
| 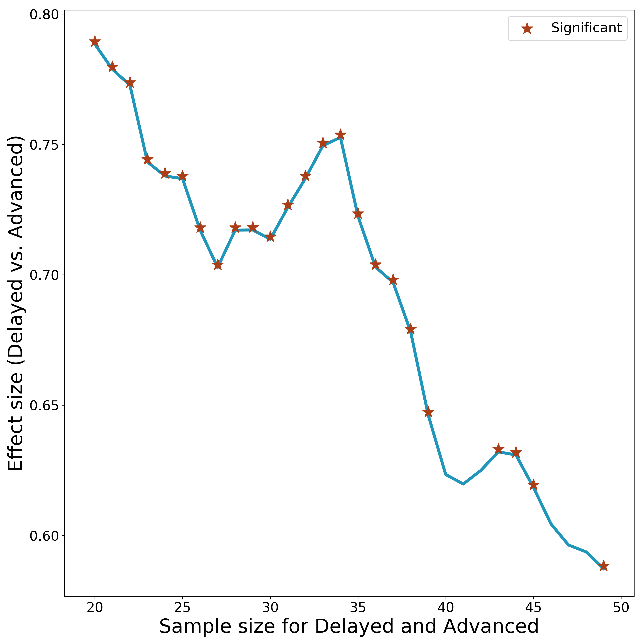  (c) | |

Figure S3. Results using Bayesian regression, with uncertainty is explicitly modeled. (a) Individuals with ASD were grouped into three subgroups based on the DDI values. Advanced group had higher brain age compared to chronological age (DDI >= 1). Delayed group had lower brain age compared to chronological age (DDI <= -1). Balanced group had similar brain age and chronological age ( -0.2 < DDI < 0.2). (b) ASD severity values for the three groups. (c) The effect size of group difference between Advanced and Delayed groups. The effect size is reported as common-language effect size (i.e., probability of having higher severity in the Delayed group), which is an appropriate choice for ordinal severity values. The effect size was calculated for varying number of people in each group (adjusting DDI threshold accordingly) to demonstrate the robustness of group difference to the DDI threshold. Regardless of the sample sizes, the inter-group difference was (almost) always significant (p < 0.05).
